# Progress in Fevers

**Published:** 1901-06-01

**Authors:** 


### Progress in Fevers.

(*Continued from page* 46.)

**Malaria.**—Lord Lister[24] reviews the history of recent researches into malaria. In 1880 Laveran discovered the malarial parasite in red blood-corpuscles, noting the elaboration of pigment and the formation of spores. Nine years later Golgi investigated the sporulating stage and found the forms different for tertian and quartan fever. He also discovered that the occurrence of fever corresponded with the maturation of the spores. Manson, observing that flagella only formed in blood after it was drawn, concluded that these bodies exercised the function of spores for the propagation of the parasite in the external world, and suggested the mosquito as an intermediate host. Ross, by a series of most patient investigations in India, on malarial parasites in man and proteosoma in birds, was able to fully establish this theory, while it remained for McCallum to explain the true function of the flagella, viz. that of spermatozoa, which, by conjunction with other granular cells—the ova—produced the vermicule.

Manson[25] points out how the many facts hitherto observed with regard to the incidence of malaria—such as association with paludal conditions, night exposure, and low altitudes—are all explained by the habits of the mosquito. Numerous recent experiments overwhelmingly prove the truth of the mosquito-malarial theory, by showing how it is possible to live with impunity in an infected district if protected from mosquito bites. Celli[26] records the striking results of his experiments among railway *employés* in Latium. He carefully excluded mosquitos from certain houses by using wire-gauze screens for the doors, windows, and chimneys; veils and gloves were worn at night out of doors. Out of 207 protected individuals 197 remained immune, although living in most malarious places. Grassi[27] conducted a similar experiment at Capaccio Fermi and Tousini[28] pursued their investigations among the convicts on the island of Asinara. A large stagnant pool was treated with kerosene twice a month and the houses were protected with gauze. No fresh case occurred as against 40 fresh cases the previous year. Sambon and Low[29] lived for four months last autumn at Ostia, one of the deadliest regions of the Campagna. They went out freely by day, but from sunset to sunrise lived in their gauze-protected house.

Grassi[30] discovered that the mosquitos which carry malarial infection all belong to the genus *Anopheles*, and of these *A. maculipennis* in Italy, *A. costalis* in West Africa, and *A. funestus* in East Africa are believed to be the species principally concerned in transmission. C. W. Daniels[31] says the last-named appears to be less strictly nocturnal in its habits than the other species. Sambon[32] gives a minute account of the life history of *A. maculipennis* in the stages of egg, larva, pupa, and perfect insect. Manson[33] shows how the mosquito breeds in pools and sluggish streams, fresh or brackish, not too densely overgrown with weeds, but provided with low forms of animal and vegetable life. E. E. Austen[34] says *Anopheles* rarely deposits her eggs in artificial collections of water, being much more particular than the ubiquitous genus *Culex*. Both larval and pupal stages are passed in water. The larva turns its head through 180° when feeding, and lies parallel with the surface. The fully-developed insect, as compared with *Culex*, a gnat of world-wide distribution, is more slender with smaller head, narrower chest and thorax, and thinner legs. In the female the palpi are quite as long as the proboscis, not mere stumps as in *Culex*. The wings of nearly all species of *Anopheles* are spotted along the costal margin. The resting position seems to vary in different species, but, as Waterhouse points out, "Whatever may be the attitude of *Anopheles*, it is all in one line; *Culex* is angular, hump-backed." The proboscis forms an angle with the body. Only the female of *Anopheles* sucks blood, and she only bites at night. A natural meal of blood is a necessary preliminary to fertilisation. One fertilisation may suffice for several batches of eggs, but a fresh meal of blood is necessary for each batch. *Anopheles* prefers the darkest and dustiest corners of houses, stables, pigstyes, and hencoops. In winter the larvæ and the perfect insect can hibernate, but, as Manson[35] and others point out, proof is wanting that the malarial parasite retains its vitality in the hibernating insect.

The malarial parasite is a small protozoan. Three species are recognised by most authorities, viz., *Hæmamœba malariæ* and *Hæmamœba vivax*, the parasites respectively of quartan and of tertian fever, belonging to the genus *Hæmamœba*; and *Hæmomenas præcox*, the parasite of pernicious or æstivo-autumual fever, belonging to the genus *Hæmomenas*. The parasite as described by D. C. Rees[36] is a tiny unicellular organism, amœbula, consisting of cell protoplasm, nucleus, and nucleolus. It moves and grows inside a red corpuscle, the hæmoglobin of which it absorbs and elaborates into melanin granules. It possesses two modes of reproduction—one by spore formation, an asexual process which takes place in man; the other, a sexual process, is only completed in the body of one genus of mosquito. The human phase of the parasite may be called the cycle of Golgi. In the blood of an infected person soon after a rigor, tiny actively amœboid bodies are found within some of the red corpuscles. These amœbulæ grow and develop pigment granules. The nucleus divides, and the pigment congregates in the centre of the amœbula, the protoplasm of which begins to segment forming a rosette-like body (sporocyte). Gradually segmentation is completed and a number of little round bodies each containing a fragment of nucleus are formed, the red cell ruptures, and the spores are set free in the blood plasma. The corpuscular *débris* and pigment granules are taken up by the phagocytes, such of the spores as escape these enter fresh red cells, and the process begins over again. In the blood of malarial patients other bodies are also found, probably developed from some of the spores. These are large pigmented spheres in the benign fevers, and crescents in the pernicious forms. The development of these in the body of the mosquito constitutes the cycle of Ross. Briefly, the changes are as follows:—If blood containing crescents be examined, the crescents are seen gradually to become spherical. Some of them become violently agitated, and shoot out one or more active, colourless, beaded flagella. These spheres are the males, their flagella are the homologues of the spermatozoa of higher animals (Ray Lankester). Other spheres—the females—remain quiet and non-flagellate. Their pigment is arranged in ring form, and they stain deeply. The next stage has only once been observed in human malaria, by McCallum in 1897. He observed a flagellum break off and enter a small papilla developed on a quiescent sphere. The cell showed some disturbance and then became elongated, developing a sharp-pointed end free from pigment. This cell is known as the travelling vermicule. It is believed to pierce the stomach wall of the mosquito and develop into a zygote. If a mosquito be examined a few minutes after feeding on a patient with crescents, flagellating and non-flagellating spheres can be seen in the blood taken into the middle intestine. Subsequent dissections show the presence of pigmented spheres (zygotes) between the muscular fibres of the intestinal wall. These grow rapidly, become encapsuled, develop smaller spheres (zygotomeres) within them, which again divide and subdivide and bud, and ultimately develop elongated sickle-shaped processes. Finally the cell bursts on its outer aspect, and these sickle-shaped bodies (sporozoites) are set free in the body fluid of the mosquito. Thence they find their way to the salivary glands, and have been traced to the end of the insect's proboscis. It is these bodies which are the actual source of infection in man, although, as Manson [37] points out, it is still unknown what determines the transformation into the intra-corpuscular body of the human phase, or into the male or female parasite. The evolution of the asexual phase of the quartan parasite occupies seventy-two hours, hence fever recurs every fourth day. In benign tertian fever the duration of the cycle is forty-eight hours. In the malignant fevers the latter half of the asexual cycle takes place in the internal organs, not in the peripheral blood. Sambon [38] believes the fever is due not to the presence of the parasites, but to the phagocytosis thereby excited, and in fact a high temperature is a favourable symptom and a low one the reverse. In tertian and quartan fevers the cold stage—during which the internal temperature may reach its maximum—is generally intense, and the hot stage slight, while sweating is profuse. As a rule these conditions are reversed in quotidian and semi-tertian types. During the cold stage the parasites are found fully developed and augmenting; during the hot stage the young organisms are attacking the erythrocytes; and during the intermission they are developing within the cells. Cases of multiple and mixed infection are very common. They often show irregular intermittent fever which tends to become continuous. Pernicious attacks are severe unchecked attacks of semi-tertian and are never first attacks. Parasites accumulate in the brain and meninges, and give rise to marked cerebral symptoms tending rapidly to coma. Sometimes they are located in the gastro-intestinal vessels, when vomiting, diarrhœa, and perhaps hæmorrhage result, and the symptoms closely simulate cholera. Typhoid and semi-tertian are also often strikingly alike. Other diseases may be present at the same time. Thus cholera in India, blackwater fever in British Central Africa, pneumonia in the Roman Campagna, siriasis in the Mississippi Valley, and polyneuritis in Jamaica may occur. These are examples of the coincidence of two diseases, not of a particular "malarial type of disease."

For treatment quinine is the only reliable drug, and Manson [39] declares that any intermittent fever which resists quinine for three or four days is not malaria. For an ordinary case 10 grains may be given when sweating commences, followed by 5 grains every six or eight hours for a week and continued at intervals for two or three months. In urgent cases it is best given by intramuscular injection or in solution by stomach or rectum. Pills, capsules, and tabloids are too insoluble for serious cases.

[24] Brit. Med. Jour., Dec. 8. [25] Practitioner, March. [26] Lancet, Dec. 1 [27] Practitioner, March, p. 266. [28] Ibid. [29] Brit. Med. Jour., Dec. 8. [30] Brit Med. Jour., Oct. 6, p. 1051. [31] Brit. Med. Jour., Jan. 26. [32] Ibid. [33] Practitioner, March. [34] Ibid. [35] Ibid. [36] Ibid. [37] Brit. Med. Jour., Dec. 15. [38] Practitioner, March. [39] Ibid.

